# Efficacy of a Brief Mindfulness Intervention in Underserved Individuals Receiving Inpatient Treatment for Opioid Use Disorder: A Pilot Study

**DOI:** 10.7759/cureus.40525

**Published:** 2023-06-16

**Authors:** Nicole R Kennelly, Suchismita Ray

**Affiliations:** 1 Health Informatics, Rutgers University, Piscataway, USA

**Keywords:** mindfulness-based stress reduction, emotion regulation, stress, drug craving, mental health, underserved population, opioid use disorder, mindfulness

## Abstract

Introduction

A mindfulness intervention is a mind-body complementary health approach that focuses on the relationships between mind, body, brain, and behavior. Mindfulness-Based Stress Reduction (MBSR) and similar mindfulness programs have been shown to decrease drug craving and relapse and improve emotional regulation, stress, pain, and anxiety. To our knowledge, a very limited number of studies have examined its efficacy in individuals from underserved populations. Underserved populations experience disparities in healthcare access, and as a result, see poorer addiction-related outcomes. The goal of this pilot study was to utilize an evidence-based, neuroscience-informed brief mindfulness intervention to improve mental health and decrease substance use behavior in a vulnerable, underserved population in New Jersey suffering from opioid use disorder (OUD).

Methods

We implemented a brief MBSR intervention in 15 underserved individuals undergoing inpatient medication-assisted treatment (MAT) for OUD. Individuals received weekly intervention sessions lasting one hour over six weeks. Furthermore, they practiced mindfulness for 10 minutes daily. Participants completed pre-and post-mindfulness intervention surveys to examine their mental well-being, drug craving, perceived stress, and emotional regulation.

Results

Within-subjects t-test results showed that compared to pre-intervention, participants showed significantly decreased perceived stress (t(14) =2.401, p=.015) and significantly decreased difficulty in emotional regulation (t(13) =3.426, p=.002 ) at post-intervention. They also showed significantly decreased drug craving post-intervention (t(14) =5.501, p=.<001). Anxiety decreased post-intervention but was not statistically significant (t(14) =1.582, p=.068).

Conclusion

This pilot study demonstrates that a brief mindfulness intervention can be effective for underserved individuals with OUD. Consistent with our hypothesis, results showed that a six-week mindfulness intervention could reduce everyday stress, drug craving, and difficulties in emotional regulation. In the future, a large-scale randomized control trial should be conducted with a control group to demonstrate the efficacy of this useful intervention.

## Introduction

The opioid crisis and overdose deaths are a result of overlapping epidemics: the long-term over-prescription and increasing dependence on opiates for pain, starting in the 2000s, an epidemic of heroin use and related deaths beginning rapid annual increases in overdose deaths in the 2010s followed by the sudden onset of epidemic-level use of synthetic opioids, and the most recent epidemic wave driven by illicitly manufactured fentanyl and stimulant use [1]. The increase in the overall substance use disorder (SUD), overdoses and fatalities across the United States (US) is driven by the dependence and misuse of opiates, contributing to 72% of all drug overdose deaths [2,3]. Nationally, the rate of drug overdose deaths continues to climb. The US surpassed 100,000 substance use-related deaths in the 12 months leading up to April 2021 [4]. While there was a 18.2% increase in the reported drug misuse and overdose deaths are on the rise for all Americans, underserved populations experience insufficient access to healthcare. Thus, underserved populations tend to be more vulnerable to the opioid crisis due to lack of healthcare access, stigma, and significant barriers to treatment not seen in more privileged groups [5,6].

FDA-approved medications to treat opioid use disorder (OUD) include methadone, buprenorphine, and naltrexone. The current treatment protocol for OUD is medication-assisted treatment (MAT) that pairs a medication for OUD with behavioral therapy. While MAT is the gold standard treatment for OUD, MAT does not adequately reduce stress-induced drug craving or the desire to use, nor does it decouple stress from craving [7-10]. Furthermore, research has demonstrated that stress, drug craving, and negative emotional affect contribute to drug relapse, opioid use, and treatment dropout for OUD patients on MAT [10-12]. Other studies suggest that an increase in stress-induced craving and poor cognitive inhibition during stress are associated with relapse factors [13]. Researchers are beginning to study interventions to implement in conjunction with MAT that address stress-induced craving and its role in impaired regulatory and reward systems in OUD. Mindfulness interventions have been studied in physical conditions such as chronic pain, psoriasis, and cancer, as well as mental health conditions including depression and anxiety [14-18]. Mindfulness has also been shown to improve burnout in healthcare workers [19]. Thus, Mindfulness-Based Stress Reduction (MBSR) and similar mindfulness interventions improve stress, craving and emotion dysregulation that MAT does not adequately address.

Research on mindfulness interventions for underserved substance users shows that the interventions are feasible, effective, and benefit this vulnerable population. A qualitative study by Spears et al. identified the importance of tailoring the traditional MBSR protocol to an underserved, non-substance-abuse population to increase the rate of success [20]. Individuals seeking mental health treatment from a community clinic participated in a focus group after two five-minute mindfulness exercises. Authors concluded that mindfulness exercises (mindful breathing and body scan) were well accepted by the non-substance-using underserved individuals; however, participants identified difficulty in finding time to meditate as a barrier to incorporating mindfulness into their life [20]. Another study by Amaro and colleagues examined a Mindfulness-Based Relapse Prevention (MBRP) intervention in minority women undergoing treatment for SUD [21]. The MBRP consisted of a nine-week intervention with 1.5- to 2-hour training sessions and a four-hour silent retreat. The researchers found a significant change in addiction severity and decreased perceived stress up to six months post-intervention. However, treatment retention and dropout rates posed issues for the study participants, suggesting a brief intervention may improve retention [1]. Another study with a single-arm cohort pilot design found an ultra-brief MBSR intervention (10- to 12-minute education session with five minutes of daily practice) to be effective in a primary care setting [22]. Thus, there is a lack of research on mindfulness interventions in underserved individuals with OUD, but there is evidence to suggest that a brief mindfulness intervention would be effective in this population.

This pilot study sought to evaluate the efficacy of a brief MBSR intervention to reduce perceived stress and craving while improving emotion regulation and anxiety symptoms in underserved individuals receiving inpatient MAT for OUD in Essex County, New Jersey; this area of New Jersey was identified in a 2020 Needs Assessment report as underserved for substance use treatment access. Essex County is made up of densely populated neighborhoods that are home to low-income individuals without sufficient healthcare access [23]. To improve treatment outcomes for these underserved opioid-using individuals, our study utilized a brief mindfulness intervention (one hour per week for six weeks), modified from the traditional eight-week Mindfulness-Based Stress Reduction intervention. We hypothesized that our brief mindfulness intervention would reduce perceived stress, opioid craving, and anxiety, and improve emotion dysregulation.

## Materials and methods

Intervention

The mindfulness intervention used in this study differed from the traditional MBSR protocol in a few ways. First, this study used the “train-the-trainer” model wherein one certified MBSR instructor trained clinicians in MBSR at an inpatient OUD treatment site. Second, MBSR is typically an in-person curriculum, but since this training occurred during the COVID-19 pandemic, the clinicians were trained on a virtual platform. Third, the trained clinicians offered a modified six-week intervention, based on the original MBSR curriculum, to their clients. The participants received one-hour mindfulness sessions with their clinicians once per week for six weeks, instead of the MBSR protocol of two-hour classes per week over eight weeks. Finally, the subjects were asked to perform 10 minutes of daily mindfulness practice on their own. This study was approved by the Rutgers Health Sciences Institutional Review Board (protocol #2021000087).

Study participants

Study participants were receiving treatment from an inpatient OUD treatment facility in New Jersey that services residents of Essex County. The inclusion criterion for the study was participation in MAT treatment due to opioid use disorder. Fifteen participants (14 male; age 25-54 years) took part in the study. Twenty percent of the participants identified as Black/African American, and 13% identified as Hispanic/Latinx. Sixty-six percent of the participants indicated that they earned less than $20,000 annually, with 93% falling below the median income for New Jersey; according to the US Census Bureau, from 2016 to 2020, the median household income of New Jersey residents was $85,254 (in 2020 US dollars) and the per-capita income was $44,153 (in 2020 dollars), putting the majority of study participants in the below-average income level group for New Jersey [24].

Data collection

After informed consent was obtained from the participants through a Zoom video conference, they completed questionnaires on demographics. Several surveys were conducted pre- and post-intervention: Perceived Stress Scale (PSS), the Beck Anxiety Inventory, Difficulties in Emotion Regulation Scale (DERS), and a craving rating. Data collection was performed on paper copies or on REDCap electronic data collection software (Research Electronic Data Capture, by Vanderbilt University).

Study variables

Several demographic variables were collected for each participant including age, gender, family annual income, race, and ethnicity. Study participants were also asked to evaluate their opioid craving in the past two weeks. Participants rated their craving on a scale from 1 (least) to 7 (most). Higher scores indicated higher opioid craving.

PSS is a 10-item assessment where respondents are asked to rate the frequency of their thoughts and feelings over the past month (i.e., is a certain thought experienced, 0 = “never”, 1 = “almost never”, 2 = “sometimes”, 3 = “fairly often”, or 4 = “very often”). Four out of the 10 items are reversed prior to being tallied, with a higher score indicative of higher perceived stress. The Beck Anxiety Inventory is a 21-item scale that lists common symptoms of anxiety and asks the subject how much they have been bothered by that symptom over the past month on a four-point scale. The items are tallied, and a higher score demonstrates more severe anxiety. DERS is a 36-item self-report Likert-type scale that asks respondents how they relate to their emotions. Higher scores suggest greater problems in emotion regulation.

Data analysis

All data was analyzed using IBM SPSS Statistics, version 28.0 (IBM Corp., Armonk, NY). First, for each participant, PSS, DERS, craving rating, and Beck Anxiety Inventory score were calculated pre- and post-intervention. Then, a within-subjects t-test was performed for PSS scores, DERS scores, craving, and Beck Anxiety Inventory scores to find how each subject compared from pre-intervention to post-intervention. While using the within-subjects t-test, each participant acted as their own control and allowed for analysis without comparison to a control group.

## Results

**Figure 1:**
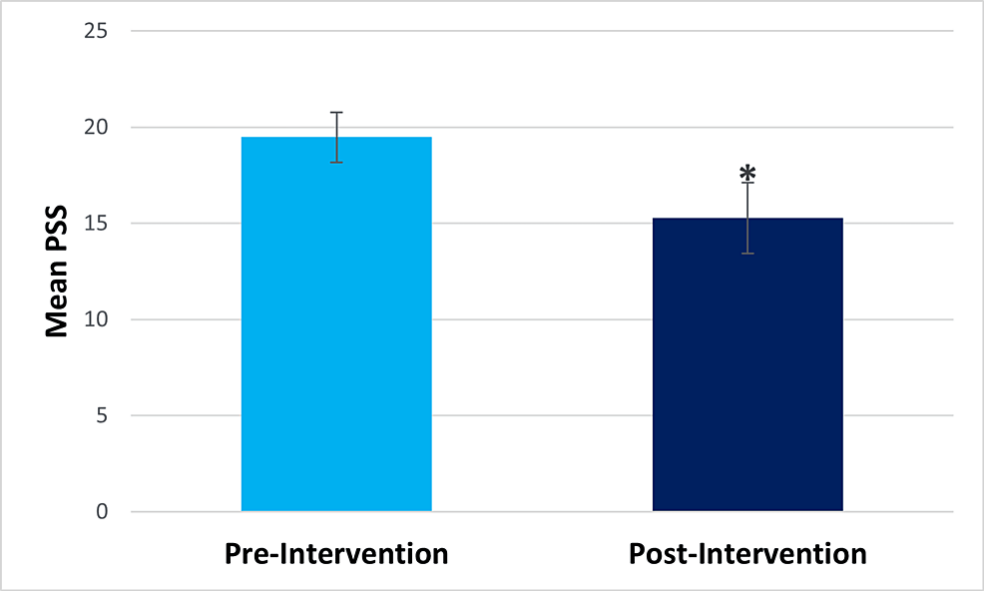
Bar graph for the mean Perceived Stress Scale (PSS) score *Statistically significant result.

**Figure 2:**
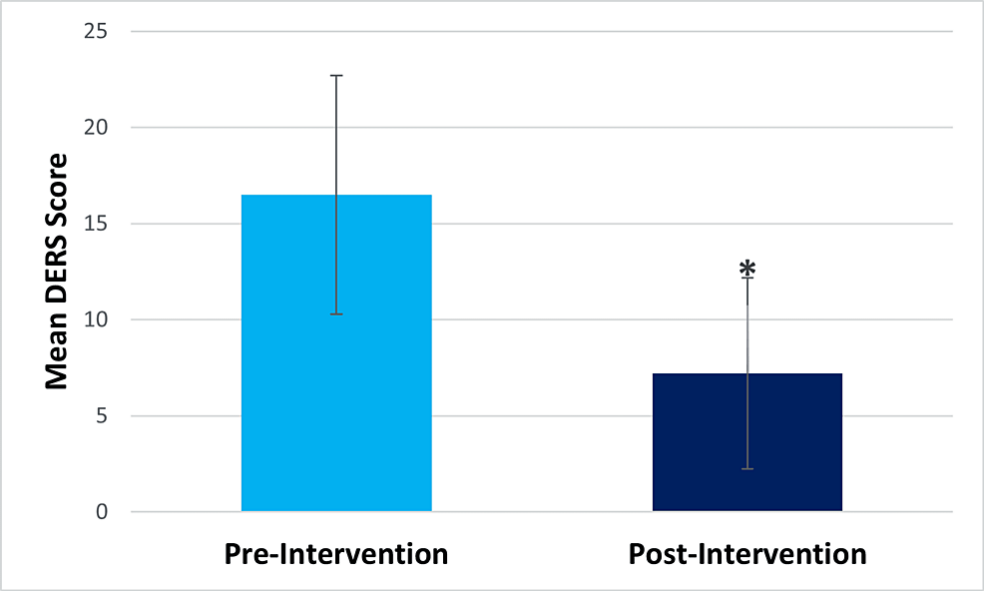
Bar graph for the mean Difficulties in Emotion Regulation Scale (DERS) score *Statistically significant result.

**Figure 3:**
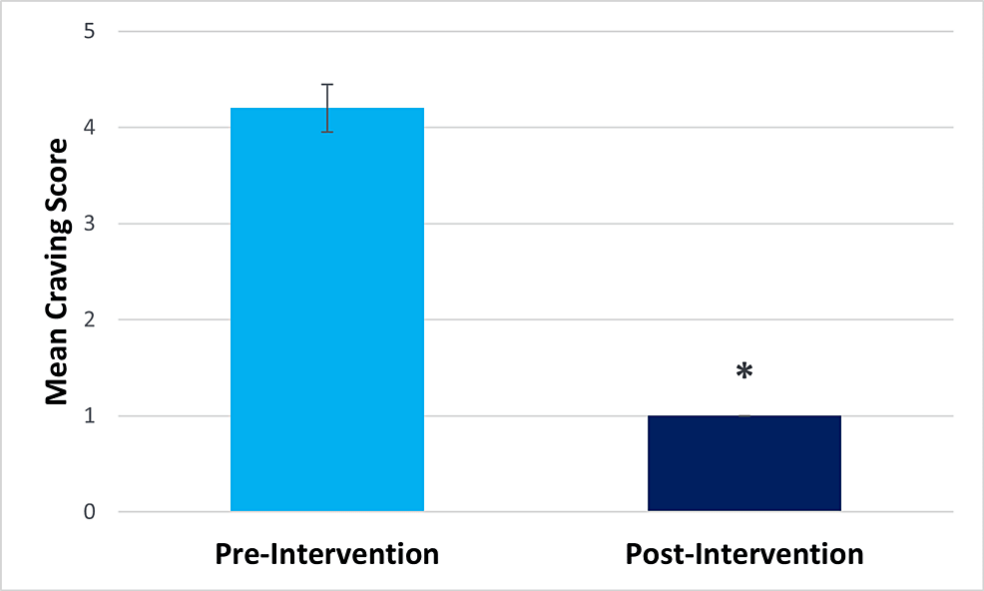
Bar graph for the mean craving score *Statistically significant result.

**Figure 4:**
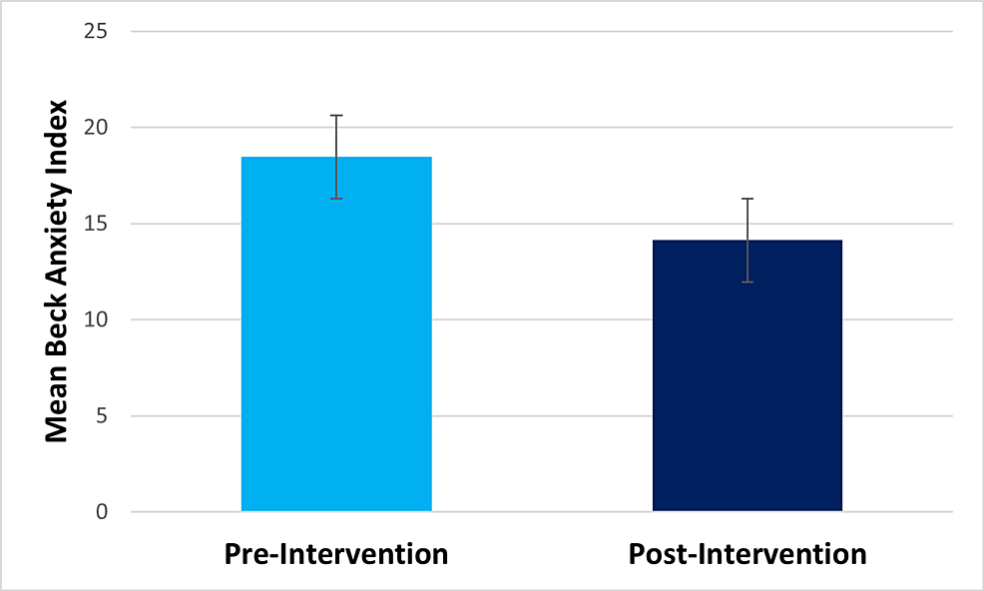
Bar graph for the mean Beck Anxiety Inventory score

Within-subjects t-test results showed that at post-intervention, compared to pre-intervention, participants showed significantly decreased perceived stress (pre-intervention mean=19.47, post-intervention mean=15.27, t(14)=2.401, p=.015) and significantly decreased difficulty in emotion regulation (pre-intervention mean=16.5, post-intervention mean=7.2, t(13)=3.426, p=.002) (Figures 1, 2, respectively). They also showed significantly decreased drug craving post-intervention (pre-intervention mean=4.2, post-intervention mean=1, t(14) =5.501, p=.<001) (Figure 3). The Beck Anxiety Inventory score was found to decrease post-intervention (pre-intervention mean=18.47, post-intervention mean=14.13) (Figure 4), but the within-subjects t-test was not statistically significant (t(14) =1.582, p=.068). For the Beck Anxiety Inventory, Cohen’s standardized mean difference (d) was .409, suggesting that the intervention had a moderate effect on anxiety and would have likely shown a significant difference between pre-and post-intervention anxiety scores if the sample size had been larger.

## Discussion

To our knowledge, this pilot study was the first to evaluate the effectiveness of a brief mindfulness intervention in underserved opioid users undergoing inpatient MAT treatment for OUD in New Jersey. We found that a six-week mindfulness intervention was effective in reducing perceived stress and opioid craving in underserved patients with OUD. The intervention also improved the patient’s ability to regulate their emotions. These findings were statistically significant and consistent with our hypothesis. Participants showed decreased anxiety post-intervention; however the reduction in anxiety was not statistically significant.

The efficacy of the original MBSR curriculum developed by Kabat-Zinn has been well documented in various clinical psychological outcomes [25]. It has been shown to decrease drug craving and relapse, and improve emotional regulation, stress, pain, depression, and anxiety [14,17,18,26-28]. Many MBSR and mindfulness studies have examined populations of generally healthy individuals or college students, but there are only a few MBSR studies that include underserved communities with SUD [29]. Also, studies in the literature evaluated the curriculum initially developed by Kabat-Zinn, which includes two-hour weekly sessions over eight weeks and includes an all-day silent retreat. While effective, the original MBSR protocol is time-intensive, and as previous studies demonstrated, time commitment to an intervention can be prohibitive for substance users and underserved populations [21]. This study that utilized a brief six-week modified mindfulness intervention based on the traditional eight-week MBSR program was effective in improving mental health symptoms and decreasing craving in underserved individuals with OUD.

Patients enrolled in our study were at least halfway through their MAT when pre-intervention questionnaires were administered, and yet, we found moderate PSS, DERS, anxiety, and craving scores at the pre-intervention timepoint. This finding is consistent with previous findings in the literature that MAT treatment alone is not sufficient for decreasing perceived stress, emotion dysregulation, and drug craving in many individuals suffering from OUD [7-9]. Our brief mindfulness intervention resulted in significant reductions in craving, perceived stress, and difficulty in emotion regulation. Although the initial study design included a control group, very few participants took part as a control, so the study proceeded as a single-arm study. We believe a single-arm study is appropriate, since MAT does not adequately address perceived stress, emotion dysregulation, or stress-induced craving.

This pilot study has a few limitations to consider. First, the number of participants included in the study was quite small (N=15), so care should be taken when extrapolating results to a larger population. Next, the study participants were admitted to an inpatient substance use treatment program, so daily stressors like food and housing security were mitigated, and our results may not be applicable to individuals experiencing homelessness or food insecurity. Additionally, since the participants had a desire to participate in the intervention, their adherence to learning mindfulness and participating in daily practice on their own may have increased. Finally, given the study participants were almost entirely male, our results cannot make inferences about how women will respond to the intervention. To the best of our knowledge, this pilot study is the first to utilize a brief mindfulness intervention involving a one-hour-long session per week for six weeks and 10 minutes of daily practice. This also appears to be the first MBSR study of an underserved population of opioid users in New Jersey. Thus, we see the benefits of a brief MBSR intervention for its ability to improve stress, opioid craving, and emotion regulation in this vulnerable, underserved population.

## Conclusions

This pilot study demonstrates the efficacy of a brief mindfulness intervention for underserved individuals in tandem with MAT in an inpatient setting. We found results consistent with our hypothesis; participants had a decrease in perceived stress and drug craving with simultaneous improvement in their ability to regulate their emotions. In future studies, we would like to further examine the efficacy of this brief intervention by performing a large, randomized control trial that would include a control group. In addition, since clinicians at the study site have now been trained to facilitate the MBSR program, we hope that they continue providing this helpful intervention to more underserved clients in New Jersey.
